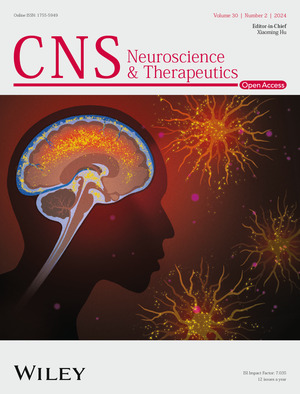# Front Cover

**DOI:** 10.1111/cns.14659

**Published:** 2024-02-29

## Abstract

The cover image is based on the Research Article *GDF15 is a dynamic biomarker of the integrated stress response in the central nervous system* by Jyoti Asundi et al., https://doi.org/10.1111/cns.14600.